# False-Positive Human Immunodeficiency Virus Results in COVID-19 Patients

**DOI:** 10.7759/cureus.34096

**Published:** 2023-01-23

**Authors:** Narek Hakobyan, Ruchi Yadav, Khaled Abaza, Adam Friedman

**Affiliations:** 1 Internal Medicine, Brookdale University Hospital and Medical Center, Brooklyn, USA; 2 Cardiology, Maimonides Medical Center, Brooklyn, USA; 3 Cardiology, SUNY (State University of New York) Downstate Medical Center, Brooklyn, USA

**Keywords:** covid-19, fourth generation hiv screening test, enzyme linked immunosorbent assay (elisa), human immunodeficiency virus infection, sars-cov-2

## Abstract

The severe acute respiratory syndrome coronavirus 2 (SARS-CoV-2) virus, which causes coronavirus disease 2019 (COVID-19) disease, was first described in 2019 and became a pandemic in 2020. Although it is possible for two viruses to co-infect together, a rarer phenomenon of false-positive results due to cross-reactivity between viruses is also possible. Herein, we present two cases of the false-positive human immunodeficiency virus (HIV) results in those infected with COVID-19. Both patients were screened for HIV and were initially found to be positive with the fourth-generation test. A subsequent blood test revealed no viral load, and an enzyme-linked immunosorbent assays (ELISA) test indicated no reactivity to HIV, thus the false initial screening test. SARS-CoV-2 is an enveloped RNA virus with its outer surface containing a spike-like glycoprotein, which allows it to recognize host cells and invade. HIV-1 gp41 and SARS-CoV-2 share several structural sequences and motifs. These similarities could explain cross-reactivity and false-positive results when screening for HIV in the presence of COVID. The presence of HIV must be confirmed through more specific laboratory tests such as ELISA.

## Introduction

Since its first description in December 2019, coronavirus disease 2019 (COVID-19) has changed the lives of millions of people worldwide. It is caused by a novel viral pathogen that is identical to that responsible for severe acute respiratory syndrome (SARS) [1,2]. Although most COVID-19 cases are asymptomatic or have only mild symptoms like cough and fever, an estimated 14% develop acute respiratory distress syndrome (ARDS) [1]. A reported 7.2% of COVID-19 cases are associated with other bacterial, fungal, or viral pathogens, which may affect survival rates and treatment options [3,4]. It is reported that some pathogens may cross-react with SARS-CoV-2 in the setting of COVID-19, suggesting a possibility of misdiagnosis [5]. The possibility of false-positive COVID-19 results should be considered in these situations since they may result in ineffective infection control and management [6]. Kliger and Levanon found that HIV and SARS-CoV proteins share sequence motifs that contribute to the construction of their active confirmations, which may explain some of their similarities [7]. Liu and colleagues have previously demonstrated that the fourth-generation antigen/antibody HIV test is susceptible to false-positive results when tested for a variety of pathogens such as the Epstein-Barr virus [8]. Other interfering substances have been reported in the literature such as rheumatoid factor, anti-hepatitis C virus, liver cirrhosis, and autoimmune disease [9]. As these cases are rare, only a few false-positive HIV results have been documented in individuals infected with SARS-CoV-2 [2,10].

Herein, we present two cases of COVID-19-positive patients who had false-positive HIV screening tests and were later confirmed to be HIV-negative. We seek to raise awareness and understand the possible reasons behind the false-positive HIV screening tests in COVID-19-positive patients and demonstrate an approach to clinical diagnosis in these patient populations.

## Case presentation

Case 1

A 69-year-old male, with a past medical history of schizoaffective disorder and hypertension, presented to the emergency department after being found unresponsive at home by his parents. The patient's baseline mental state was alert and active. In the emergency room, the patient had a poor Glasgow Coma Scale (GCS) score with diminished responsiveness to pain. Initial vital signs were; blood pressure of 157/69 mmHg, heart rate of 68 beats/min, respiratory rate of 20 breaths/min, and oxygen saturation of 99% on room air. Physical examination findings revealed an elderly male with a BMI of 21.5 kg/m^2^, intermittently responding to questions and ill-appearing. Lung auscultation revealed bilateral clear breath sounds and unlabored breathing with an appropriate rhythm. No wheezing, rales, or rhonchi were present. Cardiac auscultation yielded unremarkable findings. Neurological examination was minimal due to the patient's mental status. A resting tremor was noted on the patient's right hand.

**Figure 1:**
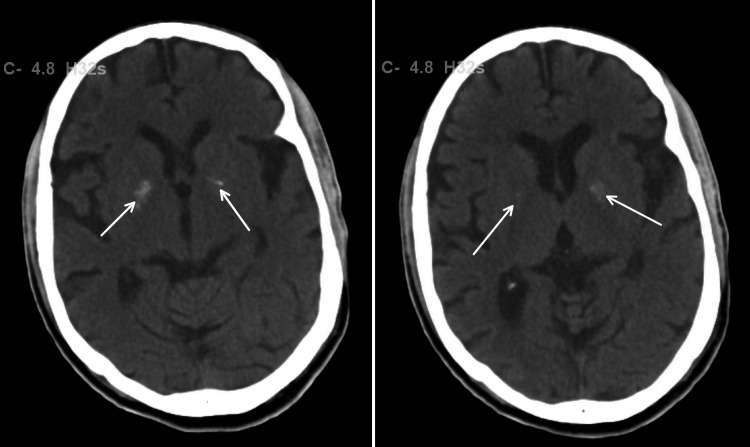
Calcification of bilateral basal ganglia

On admission, significant laboratory findings were creatinine of 1.30 mg/dL and blood urea nitrogen of 50 mg/dL. Head CT revealed bilateral basal ganglia calcification (Figure 1) with no ischemia or bleeding. X-ray findings were unremarkable. The patient was screened in the emergency department for COVID-19 viral antigen and was found to be positive, but the patient was asymptomatic. COVID-19 precautions were initiated, and the patient was isolated for treatment of acute encephalopathy secondary to the extrapyramidal side effects of antipsychotic medications with underlying COVID-19-positive infection.

Of note, the patient had an HIV test done as part of general tests for metabolic versus infectious etiology of encephalopathy. The initial test sent was an HIV combo fourth-generation test, which looks for two factors in the blood: HIV antibodies and p24 antigens. This test yielded a positive result. At the time, the patient had an absolute lymphocyte count of 458 cells/uL, an absolute CD8+ T lymphocyte count of 79 cells/uL, and an absolute CD4+ helper T-cell count of 186 cells/uL. The ratio of CD4/CD8 lymphocytes was 1.88. Bloodwork was conducted to determine genotype and viral load. The patient was immediately started on antiviral therapy with bictegravir, emtricitabine, and tenofovir alafenamide. The patient was also started on trimethoprim-sulfamethoxazole for Pneumocystis jiroveci prophylaxis. The viral load resulted in undetectable HIV viral copies. This prompted further confirmatory tests for HIV. HIV-1 genotype testing using reverse transcription-polymerase chain reaction (RT-PCR) and sequencing of the HIV-1 polymerase gene yielded non-reactive results on two different dates, one week apart. Furthermore, a follow-up antigen and antibody Western blot test also returned negative results. HIV integrase genotype was also tested in the same sample with no reactivity. The patient was concluded to have had a false positive screening test and treatment for HIV was ceased.

In the hospital, the patient was treated for COVID-19, mild acute kidney injury (AKI) secondary to rhabdomyolysis, and extrapyramidal signs secondary to antipsychotic overtreatment. Short-term rehabilitation and appropriate follow-up with consulting medical services were provided for the patient.

Case 2

An 80-year-old male with a past medical history of diabetes mellitus, hypertension, hyperlipidemia, chronic kidney disease, and Alzheimer's presented to the emergency department following a witnessed syncopal episode at home. The patient was unresponsive during the episode and regained consciousness after the arrival of emergency medical services (EMS). The patient had no bladder or bowel incontinence during the episode and no seizure activity was noted by EMS. The patient had no similar episodes in the past. The initial vital signs were blood pressure of 92/60mmHg, heart rate of 121 beats/min, respiratory rate of 19 breaths/min, oxygen saturation (SpO2) of 87% on room air, and temperature of 37.4 C. On physical examination, the patient was in no acute distress and was oriented to self and place but not to time. Pulmonary findings revealed fine crackles in the lower lung fields bilaterally. Upon deep inspiration, the patient would cough. All other system examinations were unremarkable. In the course of a routine admission screening, the patient underwent a nasopharyngeal PCR test for COVID-19, which was antigen positive.

**Figure 2:**
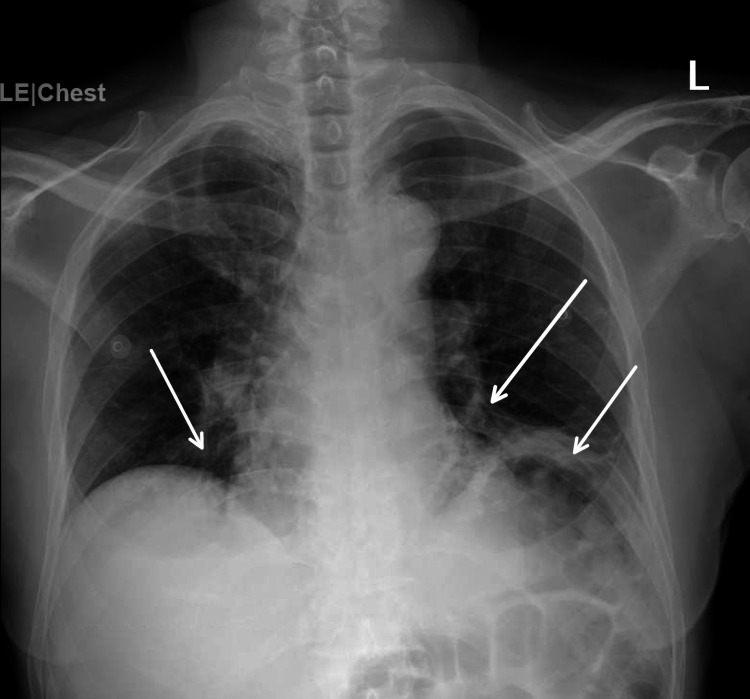
Bilateral consolidations and atelectasis of the lower lung fields

The initial significant laboratory findings were creatinine of 1.38 mg/dL, blood urea nitrogen of 31 mg/dL, white blood cell count of 12.3 x 10/L, absolute lymphocyte count of 230/uL, and absolute neutrophil count of 910/uL. Initial CT head imaging showed no acute intracranial abnormalities, age-related volume loss, and chronic white matter changes. An X-ray was performed, which showed bilateral medial lower lung pneumonia and atelectasis as shown in Figure 2. The patient was admitted and treated for COVID-19-related symptoms and AKI. 

At the time of admission, the patient was tested for HIV using a fourth-generation HIV combination test in order to exclude latent HIV as a cause of his symptoms. This test was reactive. A confirmatory enzyme-linked immunosorbent assay (ELISA) was conducted before treatment for HIV was initiated. The confirmatory test, however, yielded non-reactive results. Additionally, the viral load showed no presence of the virus (<20 copies/ml), thus confirming that the screening test was a false positive. Throughout the hospital stay, the patient received treatment for acute hypoxic respiratory failure secondary to COVID pneumonia. After being medically stabilized, the patient was discharged home.

## Discussion

COVID-19 affects multiple body organs, with the minority of cases presenting with a concurrent microbial infection, which further complicates diagnosis and treatment [3]. Studies have demonstrated that Zika and Dengue viruses can sometimes result in false-positive COVID test results [11]. It is, however, extremely rare for HIV to be mistakenly tested positive when a COVID-19 infection is present, as only a few cases have been reported [10,12].

**Figure 3:**
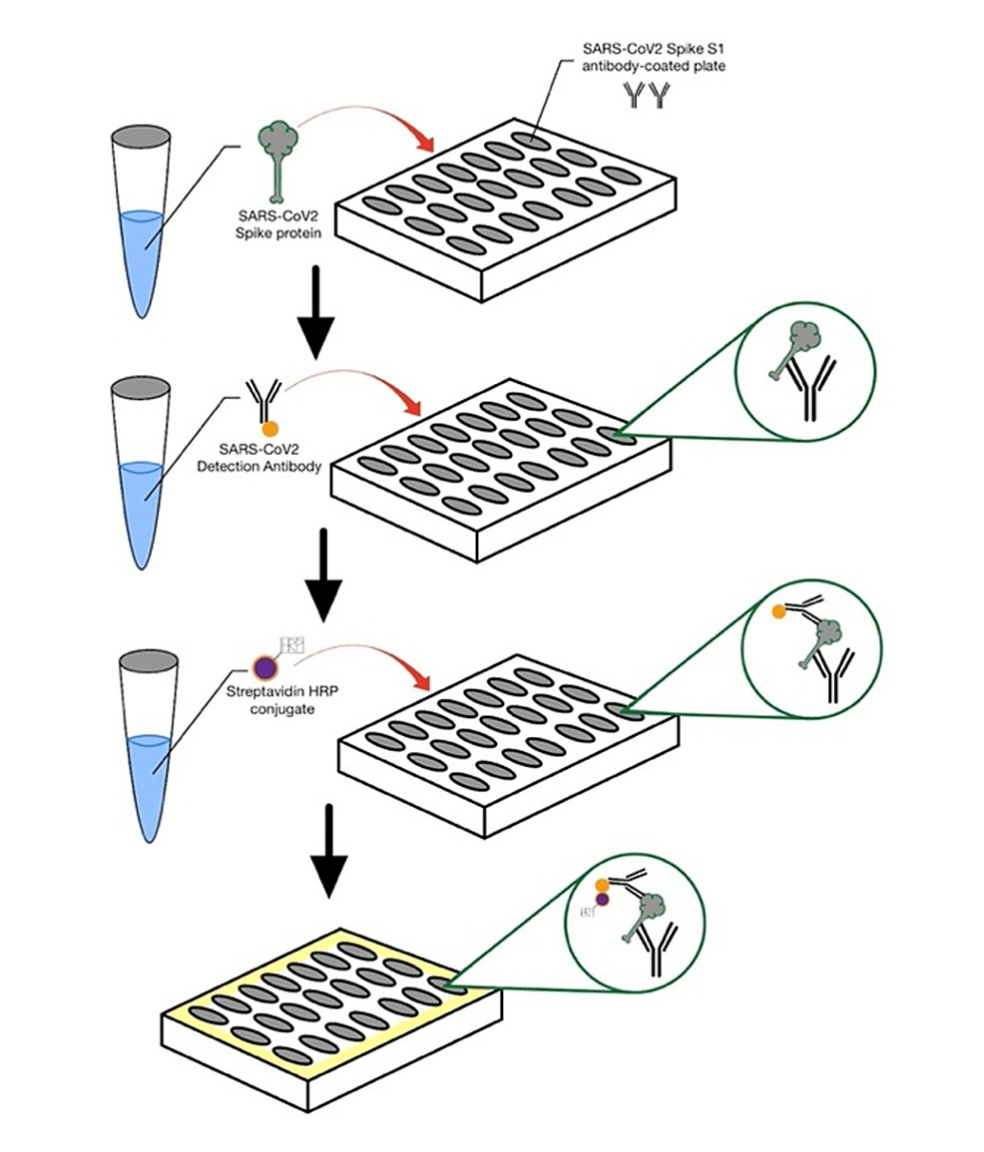
The enzyme-linked immunosorbent assay (ELISA) technique that involves the use of plate-based assay for the detection and quantification of soluble substances such as antibodies

The Coronaviridae family includes SARS-CoV-2, an enveloped RNA virus. These viruses possess transmembrane spike-like glycoproteins (S) on their outer surface, which are essential for recognizing host cells and allowing them to penetrate through. In addition, these spikes are capable of triggering humoral immunity, which stimulates the production of antibody complements [13]. Spike proteins have large surfaces and contain a receptor binding domain (RBD) and a non-RBD that promote viral particle fusion [14]. ELISA relies on the immunological principle that an antigen binds to a specific antibody, allowing the detection of very small amounts of antigens in a fluid sample such as antibodies. After binding to a specific antibody, an enzyme-coupled antibody is used to detect the antigen. Chromogenic substrate for the enzyme causes a change in fluorescence when antigen is present [15] as shown in Figure 3. The RBD domain of this spike protein has been used in ELISA to detect COVID-19 [16].

The literature suggests that the spike proteins of SARS-CoV-2 have structural similarities to some viruses, thereby making anti-body cross-reactivity between the two viruses feasible [11]. HIV-1 gp41 and SARS-CoV-2 share several structural sequences and motifs, including the N-terminal leucine/isoleucine repeat sequence and the C-terminal leucine/isoleucine repeat motif [7,17]. In addition, the helix structures of SARS-CoV-2 and HIV gp41 are very similar, suggesting that both viruses are able to fuse their membranes through the same mechanism. A spike protein present on the outer surface of SARS-CoV-2 is capable of cross-reacting with antibodies from other closely related coronaviruses. Therefore, it is possible for immunoassay tests to be adversely affected, leading to false-positive results [11]. The literature indicates that seasonal enteroviruses, such as HCoV-OC43 and HCoV-HKU1, cross-react strongly with SARS-CoV-2 [16].

The most common symptoms of COVID-19 include fever, dyspnea, and coughing [18]. HIV infection may also be associated with prolonged fevers and respiratory symptoms [12]. Laboratory tests are required to confirm the diagnosis of HIV and to determine whether the virus is present. HIV has a prevalence of 1.22% in the setting of COVID-19 [19]. In 2020, Tan et al. reported two cases of false-positive HIV diagnoses as a result of SARS-CoV-2 infection. They utilized the Abbott Architect analyzer (Abbott Laboratories, Abbott Park, Illinois) to diagnose HIV, however, immunoblot tests were negative in both cases [12]. Papamanoli and associates reported a second false-positive case in 2021 using the Abbot Architect analyzer [10]. The fourth case was described by Rawezh Salih et al. using the Roche Cobas E411 immunoassay testing platform (Roche Diagnostics, Basel, Switzerland), which resulted in a false-positive HIV test [2].

SARS-CoV-2 and HIV-1 spike proteins exhibit remarkable similarities, which may contribute to antibody cross-reactivity [20]. The incidence of false-positive HIV results in the general population is between 0.0004% and 0.0007% and depends largely on the method of diagnosis and its specificity and sensitivity [21]. HIV immunoassay tests have been reported to have an extremely high sensitivity and specificity of more than 99%. However, false results should be taken into consideration in order to avoid unnecessary or inappropriate treatment. This could result in psychological and physiological distress for patients [22]. There have also been reports in the literature that other agents, such as the Epstein-Barr virus, influenza vaccination, and the Australian COVID-19 vaccination, have caused false-positive results in HIV screening tests [21,23]. For this reason, the Australian COVID-19 vaccine was abandoned [21,23,24]. It is necessary to confirm the presence of HIV infection in cases suspected of being infected with SARS-CoV-2 by means of a more specific test, including Western Blot, Nucleic Acid (NAAT), or RNA PCR [21].

Furthermore, lymphopenia is a hallmark of HIV with significant drops correlating to worsening disease [25]. When a COVID-19-positive patient is screened, a low lymphocyte count can work to artificially confirm that a positive HIV screening test is accurate. However, Tavakolpour S et al. have demonstrated that COVID-19 infection is associated with lymphopenia [26]. COVID-19 may cause massive stress to the body, triggering lymphopenia through a stress-related mechanism involving the hypothalamic-pituitary-adrenal axis [27]. As such, lymphopenia should be evaluated for its true underlying cause.

## Conclusions

It has been over two years since COVID-19 was first observed, and as more information about the virus becomes available, its impact on medicine is continuing to evolve. The majority of research has focused on its impact on different organ systems, resulting in a variety of symptom manifestations. Herein, we present two unique cases showing the impact of COVID-19 on a diagnostic test for another disease. Clinicians should perform confirmatory HIV testing on patients who have a positive HIV screening test while being infected with COVID-19. Despite the fact that HIV chemiluminescent immunoassays have been reported to have over 99% specificity, clinicians should be aware that discordant COVID-19 and HIV results require confirmation due to the possibility that an analytical error may occur. If treatment is initiated prematurely, patient safety may be compromised.
